# Fragile X Syndrome and multidisciplinary strategy: a clinical case

**DOI:** 10.1192/j.eurpsy.2025.1549

**Published:** 2025-08-26

**Authors:** I. M. Marques, S. Abreu, M. C. Coroa

**Affiliations:** 1 Department of Psychiatry, Centro Hospitalar e Universitário de Coimbra, Coimbra, Portugal

## Abstract

**Introduction:**

Fragile X Syndrome (FXS) is a hereditary disease, linked to the X chromosome. FXS is the most common form of inherited intellectual disability and monogenic cause of Autism Spectrum Disorder (Protic *et al*. Developmental medicine and child neurology 2024; 66,7 863-871). Other clinical features includes speech and language delay, deficits in executive functioning, attention deficit hyperactivity disorder, sensory hyperarousal, social anxiety and aggressive behaviour. Cardiac abnormalities, musculoskeletal and gastrointestinal symptoms are also present.

**Objectives:**

The aim of the case is providing a review of multidisciplinar strategy in Fragil X Syndrome.

**Methods:**

Clinical case description and literature review on the subject.

**Results:**

We report a clinical case of a 25 year old man was diagnosed with autism spectrum disorder, moderate intellectual disability, associated with Fragile X Syndrome. The patient is followed up in a Psychiatry (neurodevelopment) consultation. The present clinical condition causes multiple difficulties in managing his daily life, requiring supervision to carry out most of the tasks assigned to him, resulting in dependence on a third person. Furthermore, the patient has permanent total incapacity for any employment activity. Despite the diagnosis presented, as a result of investment on the part of the clinical, school, social action teams and especially his parents, the patient does not present behavioral changes that justify, at the moment, the use of any psychotropic drug. However, it is important to have access to all non-pharmacological therapies, otherwise the patient will lose the benefits achieved to date. The patient is currently stable, taking a course to become an administrative assistant at an institution. He participates in dance groups, speech therapy and hydrotherapy.

**Conclusions:**

The best approach involves a targeted intervention to control symptoms and improving the quality of life. The management includes non-pharmacological strategies, such as individualized educational support, applied behavior analysis, physical, occupation and speech-language therapy. A pharmacologic strategies that includes, for exemple, SSRIs and/or antipsychotics is often helpful. FXS has a major impact on the individual’s social and family environment, which requires an appropriate multidisciplinary strategy that includes occupational, physical therapists, teachers, psychologists and psychiatrists. (Protic *et al.* International journal of molecular sciences 2022; 23 1935).

**Disclosure of Interest:**

None Declared

